# Deep learning-based algorithm for lung cancer detection on chest radiographs using the segmentation method

**DOI:** 10.1038/s41598-021-04667-w

**Published:** 2022-01-14

**Authors:** Akitoshi Shimazaki, Daiju Ueda, Antoine Choppin, Akira Yamamoto, Takashi Honjo, Yuki Shimahara, Yukio Miki

**Affiliations:** 1 Department of Diagnostic and Interventional Radiology, Graduate School of Medicine, Osaka City University, Osaka, Japan; 2 Smart Life Science Lab, Center for Health Science Innovation, Osaka City University, Osaka, Japan; 3 LPIXEL Inc, Tokyo, Japan

**Keywords:** Lung cancer, Software, Radiography

## Abstract

We developed and validated a deep learning (DL)-based model using the segmentation method and assessed its ability to detect lung cancer on chest radiographs. Chest radiographs for use as a training dataset and a test dataset were collected separately from January 2006 to June 2018 at our hospital. The training dataset was used to train and validate the DL-based model with five-fold cross-validation. The model sensitivity and mean false positive indications per image (mFPI) were assessed with the independent test dataset. The training dataset included 629 radiographs with 652 nodules/masses and the test dataset included 151 radiographs with 159 nodules/masses. The DL-based model had a sensitivity of 0.73 with 0.13 mFPI in the test dataset. Sensitivity was lower in lung cancers that overlapped with blind spots such as pulmonary apices, pulmonary hila, chest wall, heart, and sub-diaphragmatic space (0.50–0.64) compared with those in non-overlapped locations (0.87). The dice coefficient for the 159 malignant lesions was on average 0.52. The DL-based model was able to detect lung cancers on chest radiographs, with low mFPI.

## Introduction

Lung cancer is the primary cause of cancer death worldwide, with 2.09 million new cases and 1.76 million people dying from lung cancer in 2018^1^. Four case-controlled studies from Japan reported in the early 2000s that the combined use of chest radiographs and sputum cytology in screening was effective for reducing lung cancer mortality^2^. In contrast, two randomized controlled trials conducted from 1980 to 1990 concluded that screening with chest radiographs was not effective in reducing mortality in lung cancer^3,4^. Although the efficacy of chest radiographs in lung cancer screening remains controversial, chest radiographs are more cost-effective, easier to access, and deliver lower radiation dose compared with low-dose computed tomography (CT). A further disadvantage of chest CT is excessive false positive (FP) results. It has been reported that 96% of nodules detected by low-dose CT screening are FPs, which commonly leads to unnecessary follow-up and invasive examinations^5^. Chest radiography is inferior to chest CT in terms of sensitivity but superior in terms of specificity. Taking these characteristics into consideration, the development of a computer-aided diagnosis (CAD) model for chest radiograph would have value by improving sensitivity while maintaining low FP results.

The recent application of convolutional neural networks (CNN), a field of deep learning (DL)^6,7^, has led to dramatic, state-of-the-art improvements in radiology^8^. DL-based models have also shown promise for nodule/mass detection on chest radiographs^9–13^, which have reported sensitivities in the range of 0.51–0.84 and mean number of FP indications per image (mFPI) of 0.02–0.34. In addition, radiologist performance for detecting nodules was better with these CAD models than without them^9^. In clinical practice, it is often challenging for radiologists to detect nodules and to differentiate between benign and malignant nodules. Normal anatomical structures often appear as if they are nodules, which is why radiologists must pay careful attention to the shape and marginal properties of nodules. As these problems are caused by the conditions rather than the ability of the radiologist, even skillful radiologists can misdiagnose^14,15^.

There are two main methods for detecting lesions using DL: detection and segmentation. The detection method is a region-level classification, whereas the segmentation method is a pixel-level classification. The segmentation method can provide more detailed information than the detection method. In clinical practice, classifying the size of a lesion at the pixel-level increases the likelihood of making a correct diagnosis. Pixel-level classification also makes it easier to follow up on changes in lesion size and shape, since the shape can be used as a reference during detection. It also makes it possible to consider not only the long and short diameters but also the area of the lesion when determining the effect of treatment^16^. However, to our knowledge, there are no studies using the segmentation method to detect pathologically proven lung cancer on chest radiographs.

The purpose of this study was to train and validate a DL-based model capable of detecting lung cancer on chest radiographs using the segmentation method, and to evaluate the characteristics of this DL-based model to improve sensitivity while maintaining low FP results.

The following points summarize the contributions of this article:

- This study developed a deep learning-based model for detection and segmentation of lung cancer on chest radiographs.
- Our dataset is high quality because all the nodules/masses were pathologically proven lung cancers, and these lesions were pixel-level annotated by two radiologists.
- The segmentation method was more informative than the classification or detection methods, which is useful not only for the detection of lung cancer but also for follow-up and treatment efficacy.

## Materials and methods

### Study design

We retrospectively collected consecutive chest radiographs from patients who had been pathologically diagnosed with lung cancer at our hospital. Radiologists annotated the lung cancer lesions on these chest radiographs. A DL-based model for detecting lung cancer on radiographs was trained and validated with the annotated radiographs. The model was then tested with an independent dataset for detecting lung cancers. The protocol for this study was comprehensively reviewed and approved by the Ethical Committee of Osaka City University Graduate School of Medicine (No. 4349). Because the radiographs had been acquired during daily clinical practice and informed consent for their use in research had been obtained from patients, the Ethical Committee of Osaka City University Graduate School of Medicine waived the need for further informed consent. All methods were performed in accordance with the relevant guidelines and regulations.

### Eligibility and ground truth labelling

Two datasets were used to train and test the DL-based model, a training dataset and a test dataset. We retrospectively collected consecutive chest radiographs from patients pathologically diagnosed with lung cancer at our hospital. The training dataset was comprised of chest radiographs obtained between January 2006 and June 2017, and the test dataset contained those obtained between July 2017 and June 2018. The inclusion criteria were as follows: (a) pathologically proven lung cancer in a surgical specimen; (b) age > 40 years at the time of the preoperative chest radiograph; (c) chest CT performed within 1 month of the preoperative chest radiograph. If the patient had multiple chest radiographs that matched the above criteria, the latest radiograph was selected. Most of these chest radiographs were taken as per routine before hospitalization and were not intended to detect lung cancer. Chest radiographs on which radiologists could not identify the lesion, even with reference to CT, were excluded from analysis. For eligible radiographs, the lesions were annotated by two general radiologists (A.S. and D.U.), with 6 and 7 years of experience in chest radiography, using ITK-SNAP version 3.6.0 (http://www.itksnap.org/)*.* These annotations were defined as ground truths. The radiologists had access to the chest CT and surgical reports and evaluated the lesion characteristics including size, location, and edge. If > 50% of the edge of the nodule was traceable, the nodule was considered to have a “traceable edge”; if not, it was termed an “untraceable edge”.

### Model development

We adopted the CNN architecture using segmentation method. The segmentation method outputs more information than the detection method (which present a bounding box) or the classification method (which determine the malignancy from a single image). Maximal diameter of the tumor is particularly important in clinical practice. Since the largest diameter of the tumor often coincides with an oblique direction, not the horizontal nor the vertical direction, it is difficult to measure with detection methods which present a bounding box. Our CNN architecture was based on the encoder-decoder architecture to output segmentation^17^. The encoder-decoder architecture has a bottleneck structure, which reduces the resolution of the feature map and improves the model robustness to noise and overfitting^18^.

In addition, one characteristic of this DL-based model is that it used both a normal chest radiograph and a black-and-white inversion of a chest radiograph. This is an augmentation that makes use of the experience of radiologists^19^. It is known that black-and-white inversion makes it easier to confirm the presence of lung lesions overlapping blind spots. We considered that this augmentation could be effective for this model as well, so we applied a CNN architecture to each of the normal and inverted images and then an ensemble model using these two architectures^20^. Supplementary Fig. S1 online shows detailed information of the model.

Using chest radiographs from the training dataset, the model was trained and validated from scratch, utilizing five-fold cross-validation. The model when the value of the loss function was the smallest within 100 epochs using Adam (learning rate = 0.001, beta_1 = 0.9, beta_2 = 0.999, epsilon = 0.00000001, decay = 0.0) was adopted as the best-performing.

### Model assessment

A detection performance test was performed on a per-lesion basis using the test dataset to evaluate whether the model could identify malignant lesions on radiographs. The model calculated the probability of malignancy in a lesion detected on chest radiographs as an integer between 0 and 255. If the center of output generated by the model was within the ground truth, it was considered true positive (TP). All other outputs were FPs. When two or more TPs were proposed by the model for one ground truth, they were considered as one TP. If there was no output from the model for one ground truth, it was one FN. Two radiologists (A.S. and D.U.) retrospectively referred to the radiograph and CT to evaluate what structures were detected by the FP output. The dice coefficient was also used to evaluate segmentation performance.

### Statistical analysis

In the detection performance test, metrics were evaluated on a per-lesion basis. We used the free-response receiver-operating characteristic (FROC) curve to evaluate whether the bounding boxes proposed by the model accurately identified malignant cancers in radiographs^21^. The vertical axis of the FROC curve is sensitivity and the horizontal axis is mFPI. Sensitivity is the number of TPs that the model was able to identify divided by the number of ground truths. The mFPI is the number of FPs that the model mistakenly presented divided by the number of radiographs in the dataset. Thus, the FROC curve shows sensitivity as a function of the number of FPs shown on the image.

One of the authors (D.U.) performed all analyses, using R version 3.6.0 (https://www.r-project.org/). The FROC curves were plotted by R software. All statistical inferences were performed with two-sided 5% significance level.

## Results

### Datasets

**Figure 1:**
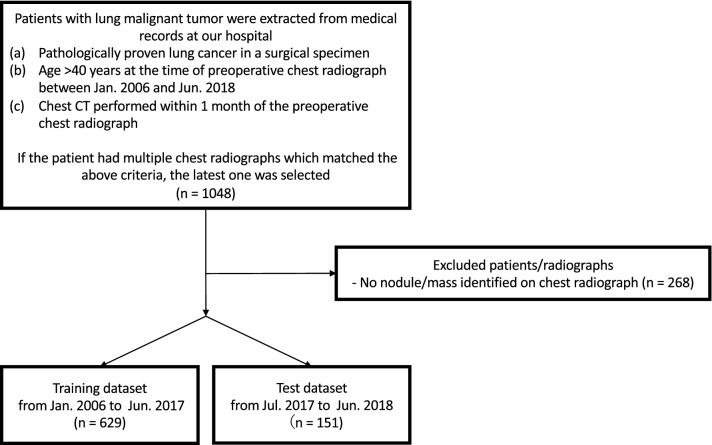
Flowchart of dataset selection.

Figure 1 shows a flowchart of the eligibility criteria for the chest radiographs. For the training dataset, 629 radiographs with 652 nodules/masses were collected from 629 patients (age range 40–91 years, mean age 70 ± 9.0 years, 221 women). For the test dataset, 151 radiographs with 159 nodules/masses were collected from 151 patients (age range 43–84 years, mean age 70 ± 9.0 years, 57 women) (Table 1).

**Table 1:** Dataset demographics.

Characteristic	Training dataset	Test dataset
**Patients (n)**	629	151
Men	408 (65%)	94 (62%)
Women	221 (35%)	57 (38%)
**Mean age ± SD (years)**		
Men	70 ± 8	70 ± 8
Women	69 ± 10	69 ± 10
Chest radiographs (n)	629	151
No. of malignant nodules/masses	652	159
Mean nodule/mass size ± SD (mm)	38 ± 21	33 ± 21
**No. of nodules/masses by size (n)**		
≤ 10 mm	5 (0.77%)	6 (3.8%)
11–15 mm	45 (6.9%)	20 (13%)
16–20 mm	68 (10%)	27 (17%)
21–25 mm	87 (13%)	23 (14%)
26–30 mm	111 (17%)	19 (12%)
31–40 mm	133 (20%)	25 (16%)
41–50 mm	73 (11%)	13 (8.1%)
> 50 mm	130 (20%)	26 (16%)
**Location of blind spot (n)**		
Total	231 (35%)	71 (45%)
Pulmonary apices	48 (7.4%)	21 (13%)
Pulmonary hila	62 (9.5%)	14 (8.8%)
Chest wall	66 (10%)	25 (16%)
Heart	39 (6.0%)	9 (5.7%)
Sub-diaphragmatic space	16 (2.5%)	2 (1.3%)
**Location of nodule/mass lesion (n)**		
Right upper	146 (22%)	40 (25%)
Right middle	147 (23%)	21 (13%)
Right lower	105 (16%)	34 (21%)
Left upper	73 (11%)	17 (11%)
Left middle	125 (19%)	36 (23%)
Left lower	56 (8.6%)	11 (6.9%)
**Margin (n)**		
Traceable edge nodule	204 (31%)	65 (41%)
Traceable edge mass	259 (40%)	60 (38%)
Untraceable edge nodule	112 (17%)	30 (19%)
Untraceable edge mass	77 (12%)	4 (2.5%)
**Pathology (n)**		
Primary lung cancer	566 (87%)	136 (86%)
Adenocarcinoma	357 (55%)	76 (48%)
Squamous cell carcinoma	156 (24%)	44 (28%)
Neuroendocrine carcinoma	26 (4.0%)	5 (3.1%)
Large cell carcinoma	4 (0.61%)	3 (1.9%)
Adenosquamous carcinoma	18 (2.8%)	1 (0.63%)
Sarcomatoid carcinoma	4 (0.61%)	7 (4.4%)
Salivary gland type carcinoma	1 (0.15%)	0 (0%)
Metastatic lung cancer	82 (13%)	23 (14%)
Malignant lymphoma	4 (0.61%)	0 (0%)

### Model test

**Figure 2:**
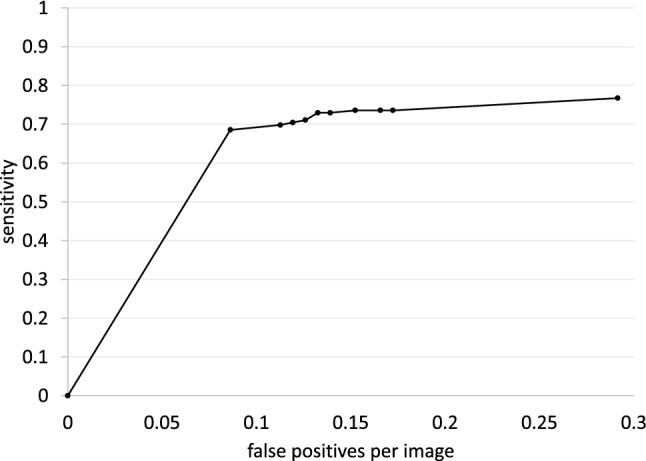
Free-response receiver-operating characteristic curve for the test dataset.

The DL-based model had sensitivity of 0.73 with 0.13 mFPI in the test dataset (Table 2). The FROC curve is shown in Fig. 2. The highest sensitivity the model attained was 1.00 for cancers with a diameter of 31–50 mm, and the second highest sensitivity was 0.85 for those with a diameter > 50 mm. For lung cancers that overlapped with blind spots such as the pulmonary apices, pulmonary hila, chest wall, heart, or sub-diaphragmatic space, sensitivity was 0.52, 0.64, 0.52, 0.56, and 0.50, respectively. The sensitivity of lesions with traceable edges on radiographs was 0.87, and that for untraceable edges was 0.21. Detailed results are shown in Table 2.

The dice coefficient for all 159 lesions was on average 0.52 ± 0.37 (standard deviation, SD). For 116 lesions detected by the model, the dice coefficient was on average 0.71 ± 0.24 (SD). The dice coefficient for all 71 lesions overlapping blind spots was 0.34 ± 0.38 (SD). For 39 lesions detected by the model that overlapped with blind spots, the dice coefficient was 0.62 ± 0.29 (SD).

**Figure 3:**
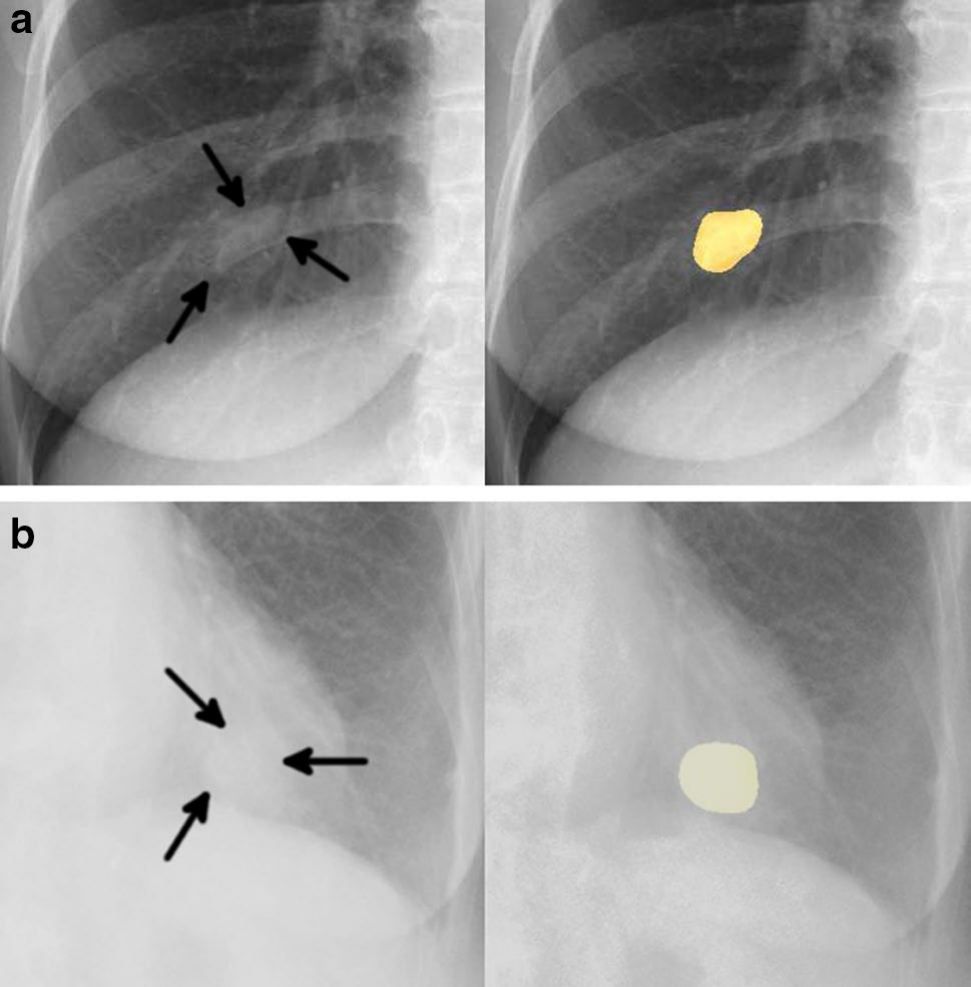
Two representative true positive cases. The images on the left are original images, and those on the right are images output by our model. (**a**) A 48-year-old woman with a nodule in the right lower lobe that was diagnosed as adenocarcinoma. The nodule was confused with rib and vessels (arrows). The model detected the nodule in the right middle lung field. (**b**) A 74-year-old woman with a nodule in the left lower lobe that was diagnosed as squamous cell carcinoma. The nodule overlapped with the heart (arrows). The lesion was identifiable by the model because its edges were traceable.

**Figure 4:**
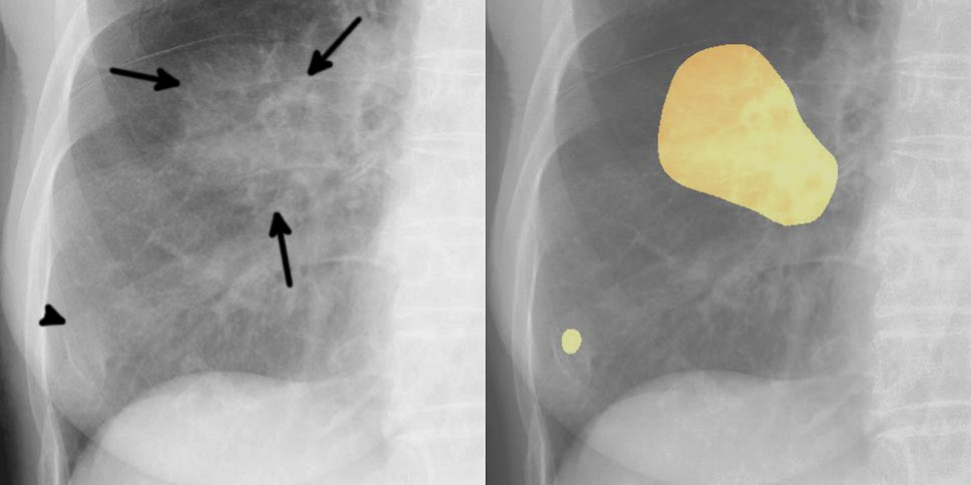
Example of one false positive case. The image on the left is an original image, and the image on the right is an image output by our model. An 81-year-old woman with a mass in the right lower lobe that was diagnosed as squamous cell carcinoma. The mass in the right middle lung field (arrows) was carcinoma. Our model detected this lesion, and also detected a slightly calcified nodule in the right lower lung field (arrowhead). This nodule was an old fracture of the right tenth rib, but was misidentified as a malignant lesion because its shape was obscured by overlap with the right eighth rib and breast.

**Figure 5:**
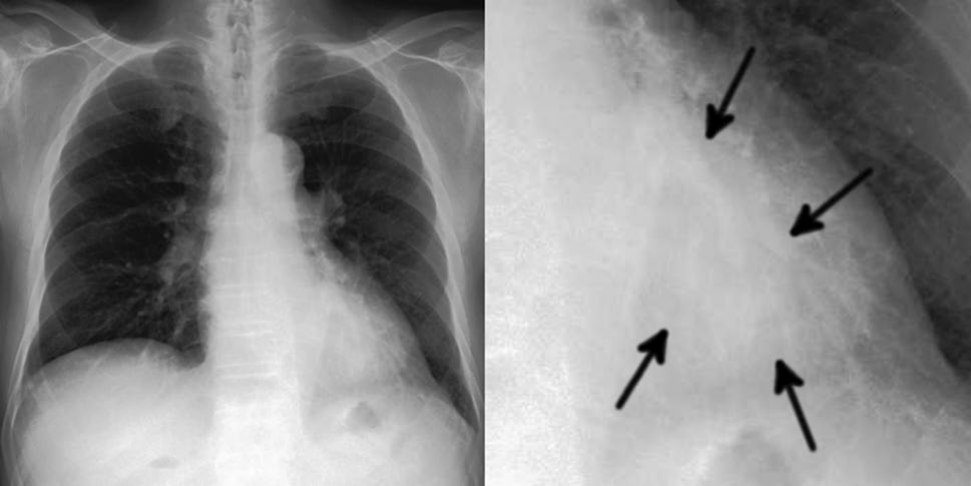
Example of one false negative case. The image on the left is a gross image, and the image on the right is an enlarged image of the lesion. A 68-year-old man with a mass in the left lower lobe that was diagnosed as adenocarcinoma. This lesion overlapped with the heart and is only faintly visible (arrows). Our model failed to detect the mass.

Of the 20 FPs, 19 could be identified as some kind of structure on the chest radiograph by radiologists (Table 3). In these 20 FPs, 13 overlapped with blind spots. There were 43 FNs, ranging in size from 9 to 72 mm (mean 21 ± 15 mm), 32 of which overlapped with blind spots (Table 4). There were four FNs > 50 mm, all of which overlapped with blind spots. Figure 3 shows representative cases of our model. Figure 4 shows overlapping of a FP output with normal anatomical structures and Fig. 5 shows a FN lung cancer that overlapped with a blind spot. Supplementary Fig. S2 online shows visualized images of the first and last layers. An ablation study to use black-and-white inversion images is shown in Supplementary Data online.

**Table 2:** Detection and segmentation performance of deep learning-based model in the test dataset.

Characteristics	Values
Total sensitivity	0.73 (0.66–0.79)
Dice coefficient ± SD	0.52 ± 0.37
**Sensitivity by size **	
≤ 10 mm	0.00 (0.00–0.00)
11–15 mm	0.38 (0.19–0.57)
16–20 mm	0.52 (0.33–0.70)
21–25 mm	0.83 (0.65–0.96)
26–30 mm	0.79 (0.58–0.95)
31–40 mm	1.00 (1.00–1.00)
41–50 mm	1.00 (1.00–1.00)
> 50 mm	0.85 (0.69–0.96)
**Sensitivity by location**	
Pulmonary apices	0.52 (0.33–0.71)
Pulmonary hila	0.64 (0.36–0.86)
Chest wall	0.52 (0.32–0.72)
Heart	0.56 (0.22–0.89)
Sub-diaphragmatic space	0.50 (0.00–1.00)
Non-overlapped lesions with normal anatomical structures	0.87 (0.79–0.93)
**Sensitivity by margin**	
Traceable edge	0.87 (0.81–0.93)
Untraceable edge	0.21 (0.06–0.35)

**Table 3:** False positive output characteristics assessed by radiologists in the test dataset.

Characteristics	No. of false positives
Total	20
**Characteristic on chest radiograph**	
Identified as some kind of structure	19 (95%)
Non-calcified nodule-like output	9 (45%)
Calcified nodule-like output	4 (20%)
Not identified any structure	1 (5.0%)
**Characteristic on CT**	
Calcified lung nodule	5 (25%)
Pulmonary artery	5 (25%)
Reticular opacity	4 (20%)
Pleural plaque	2 (10%)
Rib fracture	1 (5.0%)
Bone island	1 (5.0%)
Hilar lymph node	1 (5.0%)
Not identified any structure	1 (5.0%)
**Location of blind spots on chest radiograph**	
Total	13 (65%)
Pulmonary apices	4 (20%)
Pulmonary hila	4 (20%)
Chest wall	4 (20%)
Heart	1 (0.5%)

**Table 4:** False negative nodule/mass characteristics in the test dataset.

Characteristics	No. of false negatives
Total	43
**Size **	
≤ 10 mm	6 (14%)
11–15 mm	12 (28%)
16–20 mm	13 (30%)
21–25 mm	4 (9.3%)
26–30 mm	4 (9.3%)
31–40 mm	0 (0%)
41–50 mm	0 (0%)
> 50 mm	4 (9.3%)
**Location of blind spot**	
Total	32 (74%)
Pulmonary apices	10 (23%)
Pulmonary hila	5 (12%)
Chest wall	12 (28%)
Heart	4 (9.3%)
Sub-diaphragmatic space	1 (2.3%)
**Location of the nodule/mass lesion**	
Right upper	11 (26%)
Right middle	5 (12%)
Right lower	10 (23%)
Left upper	2 (4.7%)
Left middle	11 (23%)
Left lower	4 (4.7%)

## Discussion

In this study, we developed a model for detecting lung cancer on chest radiographs and evaluated its performance. Adding pixel-level classification of lesions in the proposed DL-based model resulted in sensitivity of 0.73 with 0.13 mFPI in the test dataset.

To our knowledge, ours is the first study to use the segmentation method to detect pathologically proven lung cancer on chest radiographs. We found several studies that used classification or detection methods to detect lung cancer on chest radiographs, but not the segmentation method. Since the segmentation method has more information about the detected lesions than the classification or detection methods, it has advantages not only in the detection of lung cancer but also in follow-up and treatment efficacy. We achieved performance as high as that in similar previous studies^9–13^ using DL-based lung nodule detection models, with fewer training data. It is particularly noteworthy that the present method achieved low mFPI. In previous studies, sensitivity and mFPI were 0.51–0.84 and 0.02–0.34, respectively, and used 3,500–13,326 radiographs with nodules or masses as the training data, compared with the 629 radiographs used in the present study. Although comparisons to these studies are difficult because the test datasets were different, our accuracy was similar to that of the detection models employed in most of the previous studies. We performed pixel-level classification of the lesions based on the segmentation method and included for analysis only lesions that were pathologically proven to be malignant, based on examination of surgically resected specimens. All previous studies^9–13^ have included potentially benign lesions, clinically malignant lesions, or pathologically malignant lesions by biopsy in their training data. Therefore, our model may be able to analyze the features of the malignant lesions in more detail. In regard with the CNN, we created this model based on Inception-ResNet-v2^17^, which combines the Inception structure and the Residual connection. In the Inception-ResNet block, convolutional filters of multiple sizes are combined with residual connections. The use of residual connections not only avoids the degradation problem caused by deep structures but also reduces the training time. In theory, the combination of these features further improves the recognition accuracy and learning efficiency^17^. By using this model with combining normal and black-white-inversion images, our results achieved comparable or better performance with fewer training data than previous studies. In regard with the robustness of the model, we consider this model to be relatively robust against imaging conditions or body shape because we consecutively collected the dataset and did not set any exclusion criteria based on imaging conditions or body shape.

 The dice coefficient for 159 malignant lesions was on average 0.52. On the other hand, for the 116 lesions detected by the model, the dice coefficient was on average 0.71. These values provide a benchmark for the segmentation performance of lung cancer on chest radiograph. The 71 lesions which overlapped with blind spots tended to have a low dice coefficient with an average of 0.34, but for 39 lesions detected by the model that overlapped with blind spots, the average dice coefficient was 0.62. This means that lesions overlapping blind spots were not only difficult to detect, but also had low accuracy in segmentation. On the other hand, the segmentation accuracy was relatively high for lesions that were detected by the model even if they overlapped with the blind spots.

Two interesting tendencies were found after retrospectively examining the characteristics of FP outputs. First, 95% (19/20) FPs could be visually recognized on chest radiographs as nodule/mass-like structures. The model identified some nodule-like structures (FPs), which overlapped with vascular shadows and ribs. This is also the case for radiologists in daily practice. Second, nodules with calcification overlapped with normal anatomical structures tended to be misdiagnosed by the model (FPs). Five FPs were non-malignant calcified lung nodules on CT and also overlapped with the heart, clavicle or ribs. As the model was trained only on malignant nodules without calcification in the training dataset, calcified nodules should not be identified in theory. Most calcified nodules are actually not identified by the model, however, this was not the case for calcified nodules that overlapped with normal anatomical structures. In other word, there is a possibility that the model could misidentify the lesion as a malignant if the features of calcification that should signal a benign lesion are masked by normal anatomical structures.

When we investigated FNs, we found that nodules in blind spots and metastatic nodules tended to be FNs. With regard to blind spots, our model showed a decrease in sensitivity for lesions that overlapped with normal anatomical structures. It was difficult for the model to identify lung cancers that overlapped with blind spots even when the tumor size was large (Fig. 5). In all FNs larger than 50 mm, there was wide overlap with normal anatomical structures, for the possible reason that it becomes difficult for the model to detect subtle density differences in lesions that overlapped with large structures such as the heart. With regard to metastatic nodules, 33% (14/43) metastatic lung cancers were FNs. These metastatic nodules ranged in size from 10 to 20 mm (mean 14 ± 3.8 mm) and were difficult to visually identify on radiographs, even with reference to CT. In fact, the radiologists had overlooked most of the small metastatic nodules at first and could only identify them retrospectively, with knowledge of the type of lung cancer and their locations.

There are some limitations of this study. The model was developed using a dataset collected from a single hospital. Although our model achieved high sensitivity with low FPs, the number of FPs may be higher in a screening cohort and the impact of this should be considered. Furthermore, an observer’s performance study is needed to evaluate the clinical utility of the model. In this study, we included only chest radiographs containing malignant nodules/masses. The fact that we used only pathologically proven lung cancers and pixel-level annotations by two radiologists in our dataset is a strength of our study, on the other hand, it may reduce the detection rate of benign nodules/masses. This is often not a problem in clinical practice. Technically, all areas other than the malignant nodules/masses could be trained as normal areas. However, normal images should be mixed in and tested to evaluate the model for detailed examination in clinical practice.

In conclusion, a DL-based model developed using the segmentation method showed high performance in the detection of lung cancer on chest radiographs. Compared with CT, chest radiographs have advantages in terms of accessibility, cost effectiveness, and low radiation dose. However, the known effectiveness of the model for lung cancer detection is limited. We believe that a CAD model with higher performance can support clinical detection and interpretation of malignant lesions on chest radiographs and offers additive value in lung cancer detection.

## Supplementary Information

Supplementary Information.
