# Supplementary material for: Deep learning-based algorithm for lung cancer detection on chest radiographs using the segmentation method
Source: Sci Rep. 2022 Jan 14;12:727. doi: 10.1038/s41598-021-04667-w (PMC8760245; doi:10.1038/s41598-021-04667-w)
Supplement: Supplementary file 1 — Supplementary Information. [file 41598_2021_4667_MOESM1_ESM.pdf]

Supplementary information

Deep learning-based algorithm for lung cancer detection on chest radiographs using the segmentation method

Akitoshi Shimazaki, Daiju Ueda, Antoine Choppin, Akira Yamamoto,

Takashi Honjo, Yuki Shimahara, Yukio Miki

a) Convolutional neural network (CNN) architecture

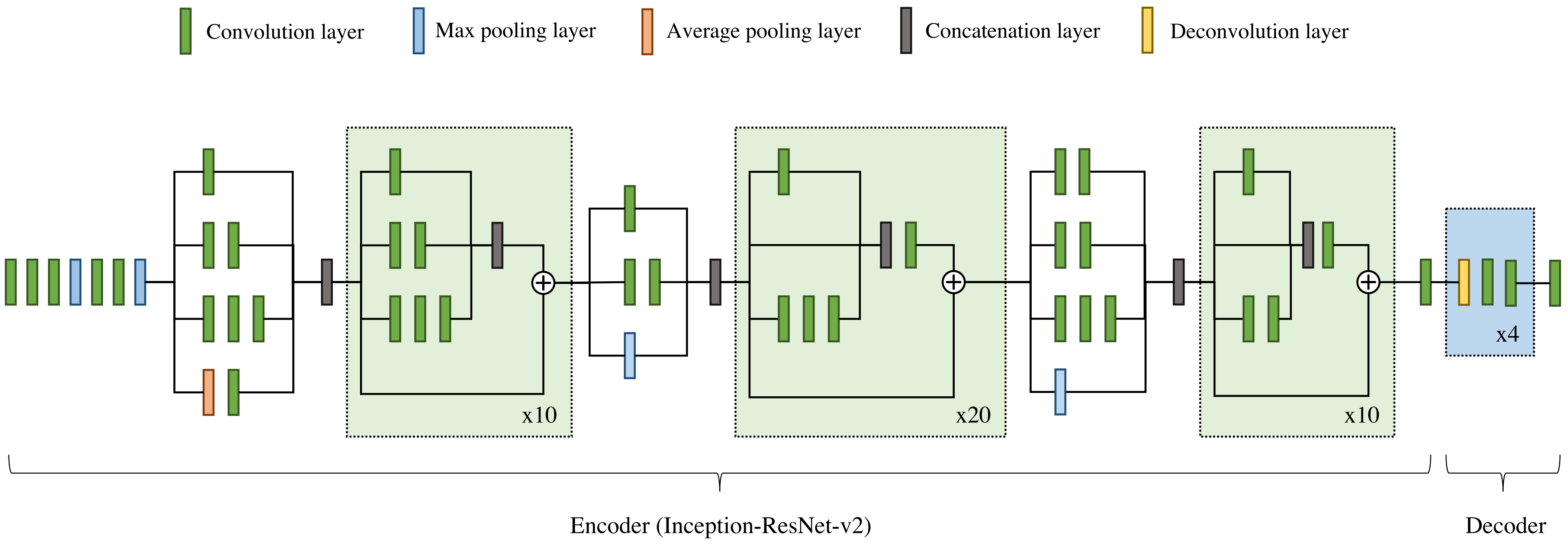

b) Deep learning (DL)-based model

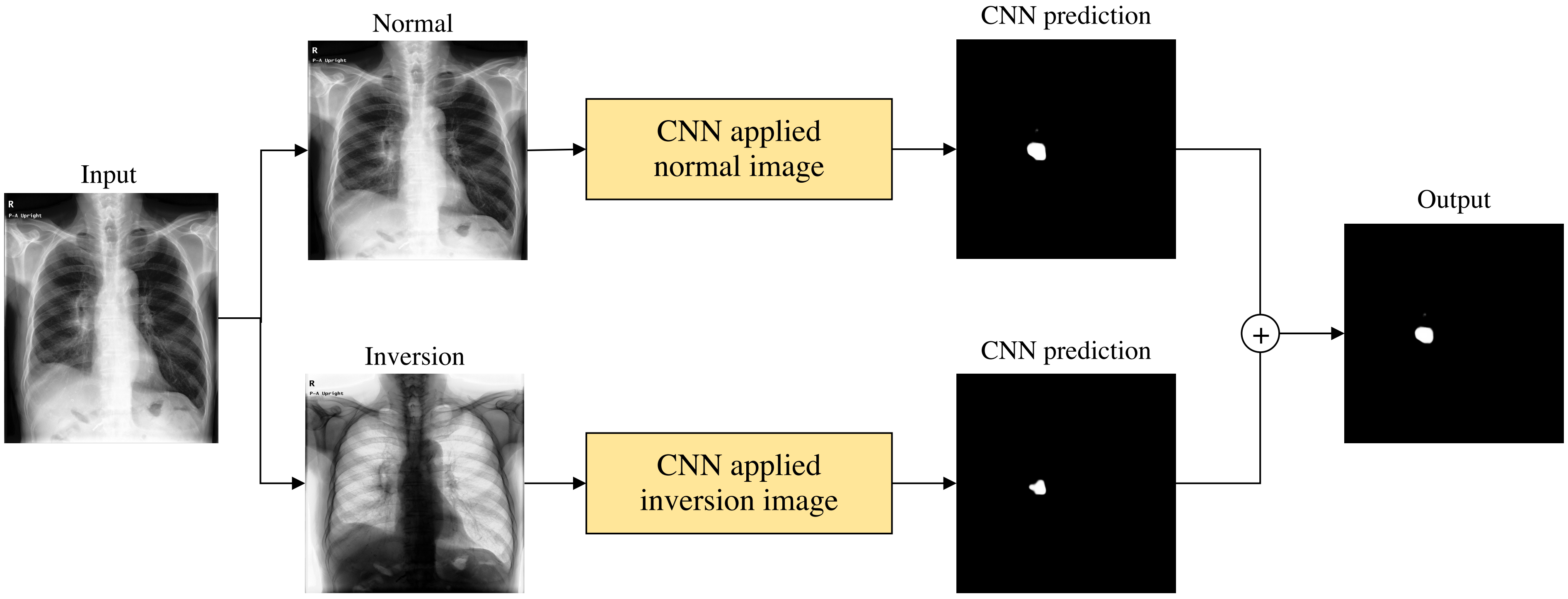

a) Our CNN architecture was based on an encoder-decoder architecture in which the encoder side extracted image features from Inception-ResNet-v2 pretrained on the ImageNet dataset. In the decoder, the features generated by the encoder side were expanded by the deconvolution layer.

b) Our DL-based model used both a normal chest radiograph and a black-and-white inversion of a chest radiograph to improve the accuracy by ensembling these models trained on these two images with each other. The input image size was reduced to 1536 × 1536. Image data augmentation was applied with random zooming, shearing, horizontal and vertical shifting, and horizontal flipping. The model was trained and validated from scratch, utilizing five-fold cross-validation. The model when the value of the loss function was the smallest within 100 epochs using Adam was adopted.

This code of Inception-ResNet-v2 is open-source software and the version we used is available from https://github.com/keras-team/keras-applications/blob/bc89834ed36935ab4a4994446e34ff81c0d8e1b7/keras_applications/inception_resnet_v2.py

Supplementary Figure S1: Model overview

a) first layer b) last layer

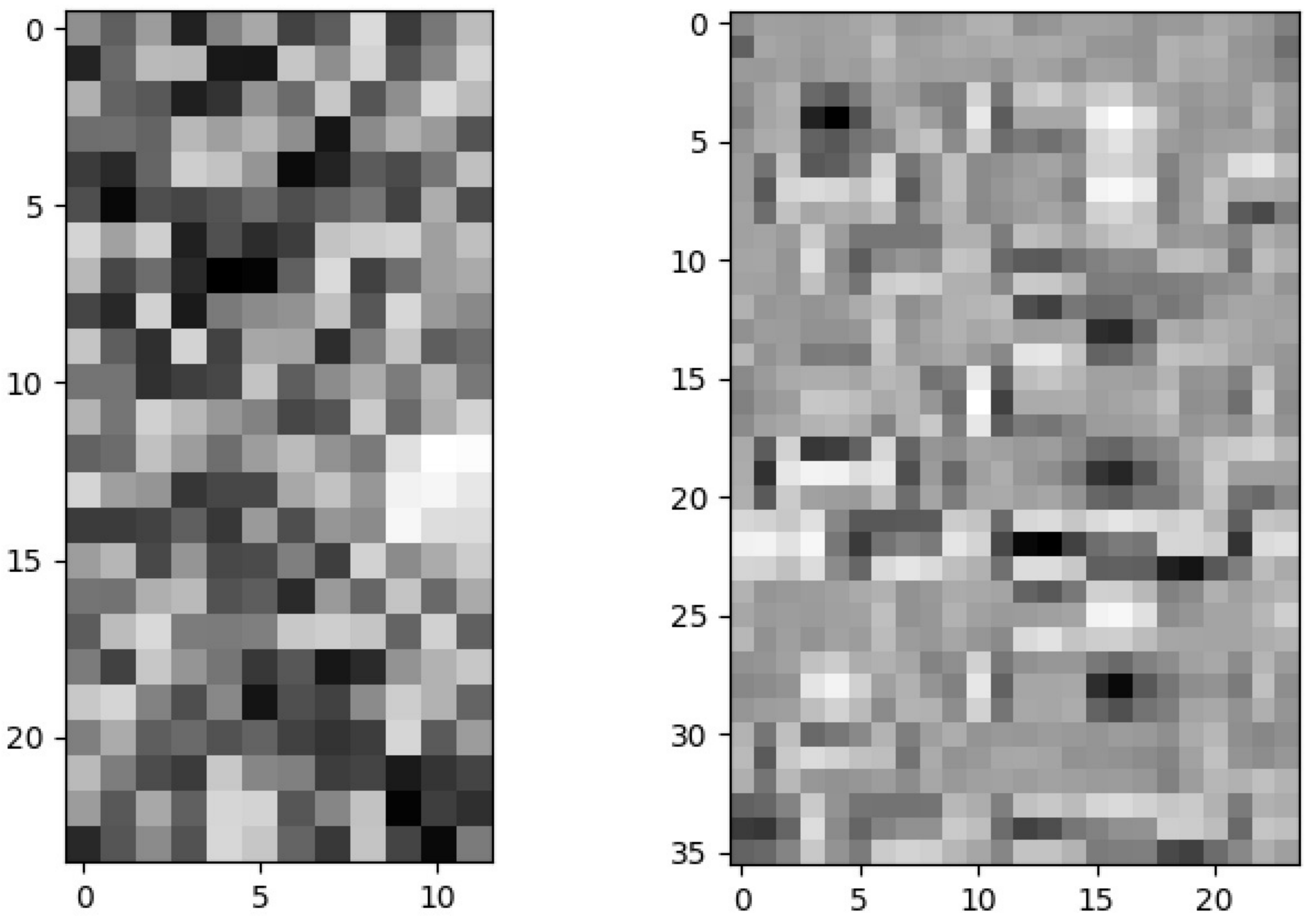

Supplementary Figure S2: Visualized images of the first and last layers in the trained model

We made the model using only normal images while the results we showed in the main body were produced by the model using both normal images and their black-white-inversion images.

Types of augmentation	Normal + Inversion	Normal only
Average dice coefficient	0.52 ± 0.37 (SD)	0.34 ± 0.34 (SD)

Supplementary Data: Dice coefficient for the ablation study validated by the model without inversion images
